# Solar Cell Parameter Extraction Method from Illumination and Dark I-V Characteristics

**DOI:** 10.3390/nano12121955

**Published:** 2022-06-07

**Authors:** Fredy Montalvo-Galicia, María Teresa Sanz-Pascual, Pedro Rosales-Quintero, Mario Moreno-Moreno

**Affiliations:** Electronics Department, National Institute for Astrophysics, Optics and Electronics, Tonantzintla 72840, PU, Mexico; materesa@inaoep.mx (M.T.S.-P.); prosales@inaoep.mx (P.R.-Q.); mmoreno@inaoep.mx (M.M.-M.)

**Keywords:** photovoltaic cells, solar cell modeling, parameter extraction, double-diode model, I-V characteristics

## Abstract

A novel method to extract the seven parameters of the double-diode model of solar cells using the current–voltage (I-V) characteristics under illumination and in the dark is presented. The algorithm consists of two subroutines which are alternatively run to adjust all the parameters of the cell in an iterative process. Curve fitting of the light I-V characteristics ensures accuracy in the prediction of the maximum power point, whereas simultaneously fitting the dark I-V characteristics results in a set of physically meaningful parameters that provide information about the physical performance of the photovoltaic devices. Experimental I-V curves of in-house solar cells are used to validate the proposed parameter extraction method, which can be furthermore applied to other types of p–n junction-based photovoltaic devices.

## 1. Introduction

Parameter extraction is a fundamental process to evaluate the performance of photovoltaic (PV) devices. The obtained parameters can be used not only to predict the behavior of solar cells but also to obtain essential information about device performance and efficiency. The single-diode and double-diode models (SDM and DDM) are most commonly used to describe the I-V characteristic of solar cells through the Shockley equation, and they include series and shunt resistances to account for the current-dependent and voltage-dependent loss mechanisms [1,2]. However, the SDM does not accurately model the physical phenomena in the solar cell, as it does not take into account the recombination process [3,4]. The DDM, in turn, is a more precise model, and it provides the necessary insight into the physics of the solar cell with moderate complexity [5,6,7]. Even though more complex models exist, such as the three-diode model, their use is limited by the computational complexity [1].

The data set used to extract the parameters of solar cells should be carefully chosen. The dark current–voltage (I-V) characteristic is more sensitive than light I-V characteristics to determine the DDM parameters [8]. However, most often, the parameter extraction process is carried out from the illumination case, as the main goal of the model is usually to predict the maximum power point (MPP) in order to analyze the dynamic behavior of MPP tracking (MPPT) algorithms [3,9]. As a result, the obtained parameter set is useful in these kinds of simulations, but it may fail in predicting the electrical performance of the photovoltaic device. In some cases, the parameters of the solar cells are extracted, taking into account only the dark I-V characteristic [10,11,12,13,14,15,16], which can lead to high inaccuracy in the prediction of the MPP under illumination conditions, as the dark I-V measurement procedure does not provide information regarding short-circuit current [8].

There are mainly three types of parameter extraction methods: explicit, iterative and based on optimization [17,18]. The explicit (or analytical) methods are fast, as they use simplifications and empirical observations in order to obtain a set of equations that describes the cell behavior and can be solved without an iteration process [18,19,20,21]. However, the approximations made, which sometimes include neglecting the series and/or parallel resistances, or considering the saturation currents of both diodes in the DDM to be equal, lack accuracy and may lead to unrealistic solutions [2,18,22]. Furthermore, the accuracy of the explicit models depends on the photovoltaic technology [23]. The optimization methods, in turn, use soft computing techniques to extract the cell parameters, which results in high accuracy at the cost of computational complexity [24,25]. In this paper, to obtain a good trade-off between complexity and accuracy, an iteration process is proposed which, as often done, starts with an initialization step based on simplified equations. The proposed algorithm is divided into two subroutines to fit both the illumination and the dark I-V characteristics, which are alternatively run. This results in a more meaningful parameter set, which can be used both to predict the MPP and to obtain information with regard to the physical performance and characteristics of the PV device. Furthermore, the proposed parameter extraction method can be applied to other types of photovoltaic devices based on the p–n junction, such as organic or heterojunction with intrinsic thin-layer (HIT) solar cells, either commercial or fabricated in research laboratories [26,27].

In fitting the illumination I-V characteristic, the ideality factors of the diodes in the model are often initially set at a constant value and not further adjusted [5,28,29,30]. The ideality factor indicates how closely the properties of the diode match the ideal properties, and it approaches n=2 if the recombination process dominates. A bad choice of this value in the parameter extraction process can seriously affect the accuracy of the model or provide parameter values that lead to good fitting of the I-V characteristic but lack physical meaning [31,32]. In the hybrid method proposed by Chennoufi et al. [31], the sum of $n_{1}$ and $n_{2}$ is assumed to be higher than or equal to three, based on [33], and two iterative processes, the first one to adjust the series resistance and the second one to adjust $n_{1}$ and $n_{2}$, are carried out. In Tifidat et al. [32], the ideality factors and the series resistance are found in the same iterative process, and the rest of parameters are calculated analytically. The values of $n_{1}$ and $n_{2}$, which are supposed to be close to 1 and 2, respectively, are initialized in each iteration. In Chin et al. [7], where an analytical and an optimization method are combined, the searching space for the ideality factors is $n_{1}\in \left[1,2\right]$ and $n_{2}\in \left[2,4\right]$, whereas in [34], based on analytical method, $n_{1}$ is set to 1 and $n_{2}\in \left[1.2,2\right]$. In Orioli et al. [35], the fitting procedure is nested in a final iteration in which $n_{2}$ is gradually reduced to find the smallest value that still results in positive values of all the other parameters, with $n_{2}>n_{1}$. The value ranges in each paper are therefore quite different and often times not justified. In this work, the ideality factors are initialized to $n_{1}=1$ and $n_{2}=1.3$, whereas the initial values of the remaining parameters are analytically calculated. Then, a curve-fitting algorithm is applied in which all of them, including the ideality factors, are adjusted in each iteration.

As for the parameter extraction from the dark I-V characteristic of solar cells, it is usually carried out by dividing the logarithmic plot into three different regions, where the curve is dominated either by the series resistance, the shunt resistance or the diode effects [10,12,13,14,15,36]. A similar procedure will be carried out here, although in this case, the characteristic is divided into four regions to adjust the required six parameters.

The paper is organized as follows. Section 2 describes the proposed parameter extraction method, which consists in alternatively running an illumination I-V curve fitting and a dark I-V curve fitting subroutine. In Section 3, the proposed algorithm is applied to three different in-house fabricated solar cells in order to validate the accuracy of the method. Finally, conclusions are drawn in Section 4.

## 2. Parameter Extraction Algorithm

**Figure 1:**
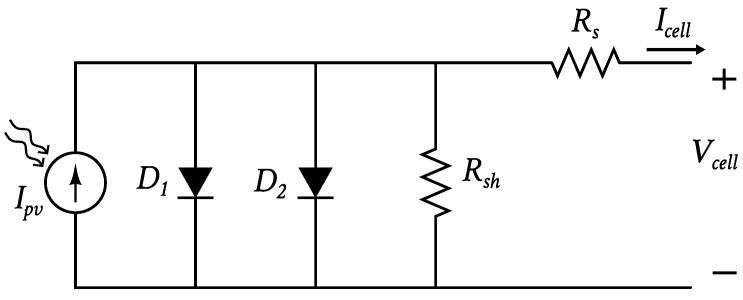
Double-diode solar cell model (DDM).

The DDM consists, as shown in Figure 1, of two diodes, $D_{1}$ and $D_{2}$, which model the carrier diffusion and the carrier recombination process, respectively, a series resistance $R_{s}$, which represents the internal losses, and a shunt resistance $R_{sh}$, which represents the leakage current in the p–n junction [5,37,38]. It requires seven parameters: the photo-generated current $I_{pv}$, the saturation current $I_{si}$ and ideality factor $n_{i}$ of diode $D_{i}$ (with i=1,2), the shunt resistance $R_{sh}$, and the series resistance $R_{s}$.

The output current $I_{cell}$ is obtained by applying the Kirchhoff’s current law:

(1) $$I_{cell}=I_{pv}-I_{s1}\left[exp\left(\frac{V_{cell}+I_{cell}R_{s}}{n_{1}V_{t}}\right)-1\right]-I_{s2}\left[exp\left(\frac{V_{cell}+I_{cell}R_{s}}{n_{2}V_{t}}\right)-1\right]-\left(\frac{V_{cell}+I_{cell}R_{s}}{R_{sh}}\right)$$

where $V_{t}$ is the thermal voltage, and $V_{cell}$ corresponds to the solar cell voltage. Equation (1) is valid both under illumination and under dark conditions (with $I_{pv}=0$), and it does not have an easy analytical solution.

The proposed parameter extraction algorithm uses the I-V characteristics under illumination and in the dark, so it can predict the MPP and, at the same time, give more insight into the physical structure of the solar cell, as the accuracy in predicting the characteristics under both conditions is improved. In particular, the proposed parameter extraction algorithm is divided into two subroutines: IIVf (illumination I-V fitting) and DIVf (dark I-V fitting), which are alternatively run until good fitting in both conditions is achieved. The IIVf subroutine is first executed with the experimental input data: short-circuit current $I_{sc}$, open-circuit voltage $V_{oc}$, maximum power point voltage $V_{mpp}$, and current $I_{mpp}$. The output file with the extracted parameter set is then fed into the DIVf subroutine, which adjusts the cell parameters so as to fit the experimental dark I-V characteristic. The new output file with the updated parameter set is then fed into the IIVf subroutine, and the process is repeated until a minimum difference between parameters is found at the end of subsequent subroutines. The goal precision is set by the user at the beginning of the process. A full iteration consists in the successive execution of the IIVf and DIVf subroutines. The final parameter set is selected in terms of the lowest Root Mean Square Error (RMSE) values. Each subroutine is explained next.

### 2.1. Illumination I-V Fitting (IIVf) Subroutine

**Figure 2:**
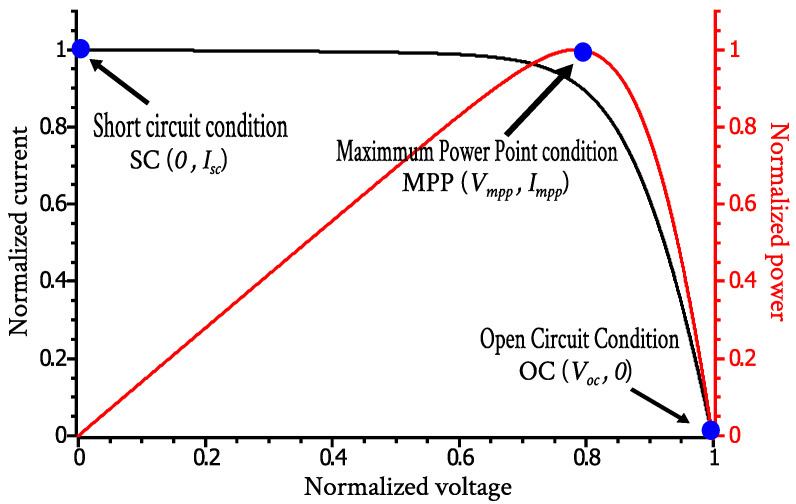
Normalized I-V and P-V characteristics of a solar cell.

From the typical I-V characteristics of the solar cell, shown in Figure 2, three main points can be extracted: the short circuit (SC) condition, where $I_{cell}=I_{sc}$ and $V_{cell}=0$, the open circuit (OC) condition, where $I_{cell}=0$ and $V_{cell}=V_{oc}$, and the MPP condition, where $I_{cell}=I_{mpp}$ and $V_{cell}=V_{mpp}$. The first time the IIVf subroutine is run, the value of the parameters is estimated before the iteration process from these three points, which divide the I-V characteristic into two regions: between the SC condition and the MPP, the solar cell approximately behaves as a current source, whereas between the MPP and the OC condition, it approximately works as a voltage source. The slope of the I-V characteristic in these regions provides an initial estimation of the $R_{s}$ and $R_{sh}$ resistance values [4], which can be approximated as:

(2) $$R_{sh}=\frac{V_{mpp}}{I_{sc}-I_{mpp}}$$

(3) $$R_{s}=\frac{V_{oc}-V_{mpp}}{I_{mpp}}$$

The value of the $I_{pv}$ current, in turn, is approximately given by [3]:

(4) $$I_{pv}=\frac{R_{s}+R_{sh}}{R_{sh}}I_{sc}$$

The ideality factors are initialized to their lowest value: $n_{1}=1$ and $n_{2}=1.3$. The initial value of the saturation current $I_{s1}$ of $D_{1}$ is determined from the approximation used in [3]:

(5) $$I_{s1}=\frac{I_{sc}}{exp\left(\frac{V_{oc}}{n_{1}V_{t}}\right)-1}$$

where the open circuit condition is used in Equation (1) and the contribution of resistors is neglected.

As for the saturation current $I_{s2}$ of the recombination diode, it is known to be at least two or three orders of magnitude higher than $I_{s1}$[5]. In this work, $I_{s2}$ current is initially approximated, after neglecting the effect of diode $D_{1}$ and the series and shunt resistances, as:

(6) $$I_{s2}=\frac{I_{sc}}{exp\left(\frac{V_{oc}}{n_{2}V_{t}}\right)-1}$$

**Figure 3:**
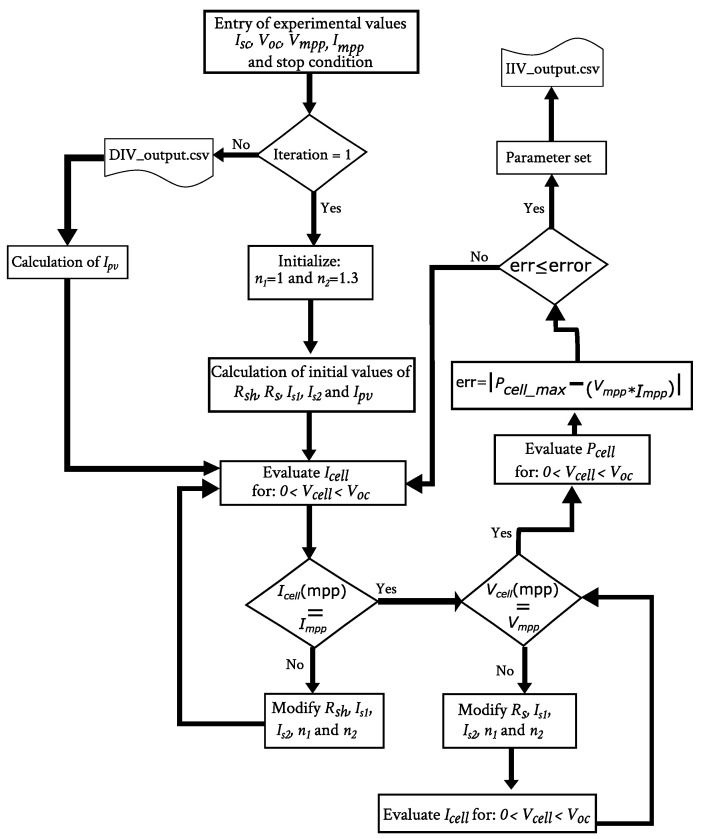
Flow diagram of the IIVf subroutine.

The following iteration process is based on the dependence of the illumination I-V characteristic on each parameter, as given by Equation (1). Table 1 summarizes how the maximum power changes when any of the parameter changes, at constant $I_{sc}$ and $V_{oc}$ values. A graphical illustration of these dependencies can be found in references [1,2]. So, after initializing the parameter values, the iteration process in Figure 3 begins. The adjustments are performed according to Table 1 and, in contrast to other parameter extraction methods, all the parameters can be modified, if necessary, in each iteration. In particular, the ideality factors are set within the ranges: $n_{1}\in \left[1,1.3\right]$ and $n_{2}\in \left[1.3,2\right]$. The stop condition is the maximum error allowed in the prediction of the maximum power $P_{max}$. This error is set by the user and in our case was chosen to be lower than 1%. Once the resulting parameter set is obtained, the values are fed into the DIVf subroutine.

### 2.2. Dark I-V Fitting (DIVf) Subroutine

The current of the solar cell in the dark is obtained from Equation (1) with $I_{pv}=0$:

(7) $$I_{cell}=I_{s1}\left[exp\left(\frac{V_{cell}-I_{cell}R_{s}}{n_{1}V_{t}}\right)-1\right]+I_{s2}\left[exp\left(\frac{V_{cell}-I_{cell}R_{s}}{n_{2}V_{t}}\right)-1\right]+\left(\frac{V_{cell}-I_{cell}R_{s}}{R_{sh}}\right)$$

**Figure 4:**
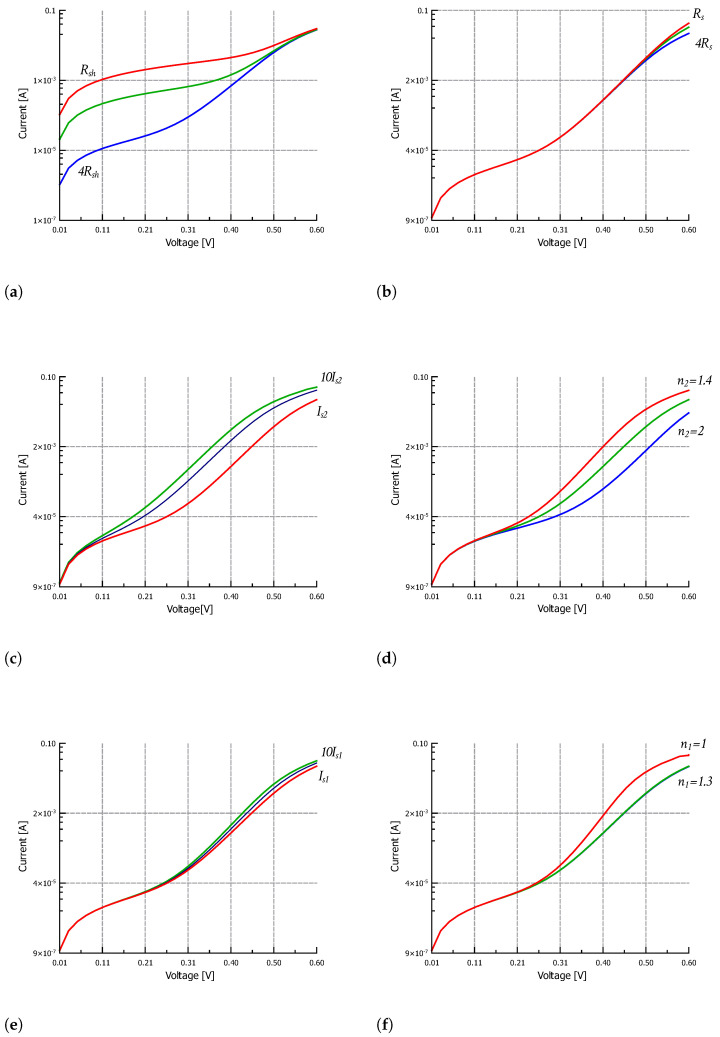
Dark I-V characteristic variation for: (**a**) $R_{sh}$ variation, (**b**) $R_{s}$ variation, (**c**) $I_{s2}$ variation, (**d**) $n_{2}$ variation, (**e**) $I_{s1}$ variation and (**f**) $n_{1}$ variation.

From the logarithmic plot of the I-V characteristic, four different regions can be identified where the curve is dominated by different loss mechanisms. Figure 4 shows how the characteristic changes when each parameter is varied. At low voltages, the current is limited by the shunt resistance: the higher the $R_{sh}$ value, the lower the reverse current, as shown in Figure 4a. At high voltages, the series resistance limits the maximum current: the higher the $R_{s}$ value, the lower the saturation current, as shown in Figure 4b. In the lower intermediate voltage range, the effect of the recombination diode $D_{2}$ dominates, and in particular, $I_{s2}$ shows the highest impact on the I-V characteristic (Figure 4c). In contrast, the most dominant parameters in the upper intermediate voltage range are $n_{2}$, $I_{s1}$ and $n_{1}$ of the diffusion diode $D_{1}$ (Figure 4d–f).

**Figure 5:**
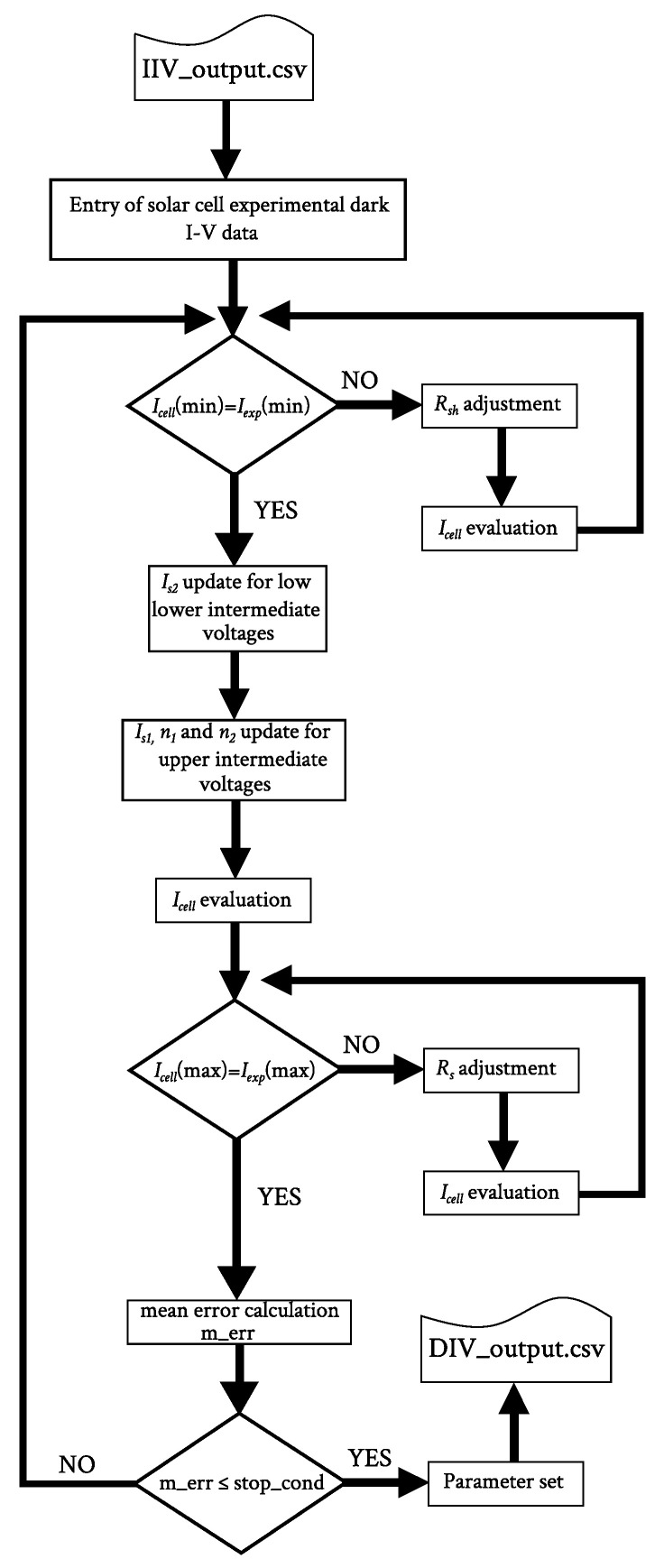
Flow diagram of the DIVf subroutine.

The DIVf subroutine, whose flow diagram is shown in Figure 5, starts by initializing the parameter values to the parameter set provided by the IIVf subroutine. Then, the resulting dark I-V characteristic is compared with the experimental data in four steps, corresponding to the four aforementioned voltage ranges, and the corresponding parameters are adjusted in each step if necessary. $R_{sh}$ and $R_{s}$ are adjusted in accordance to the minimum and maximum output current, respectively, whereas $I_{s2}$, $n_{2}$, $I_{s1}$ and $n_{1}$ are adjusted, taking into account the slope of the I-V characteristic and the mean value of the output current in the intermediate voltage ranges.

The parameters obtained from this subroutine are used as initial values for the IIVf subroutine. So, the process is repeated until the difference between the parameters obtained from subsequent IIVf and DIVf subroutines is minimum. The stop condition must be established by the user. In the end, the valid parameter set is chosen to be the one with the lowest RMSE, i.e., the one that best fits both the illumination and dark characteristics.

## 3. Parameter Extraction of Crystalline Solar Cells

**Figure 6:**
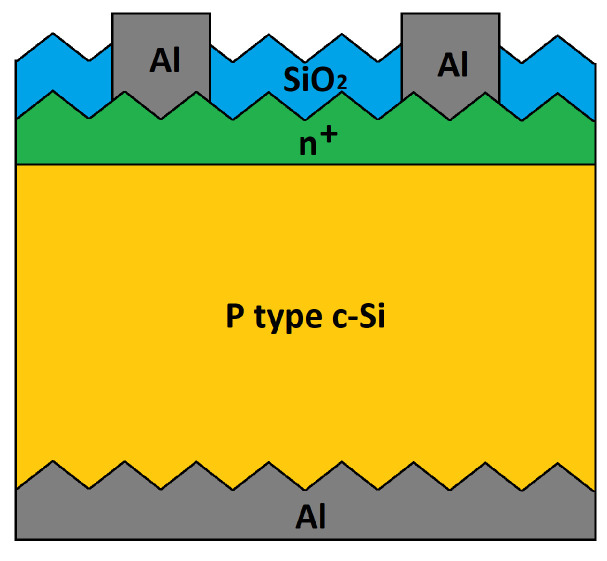
Fabricated solar cell structure.

**Figure 7:**
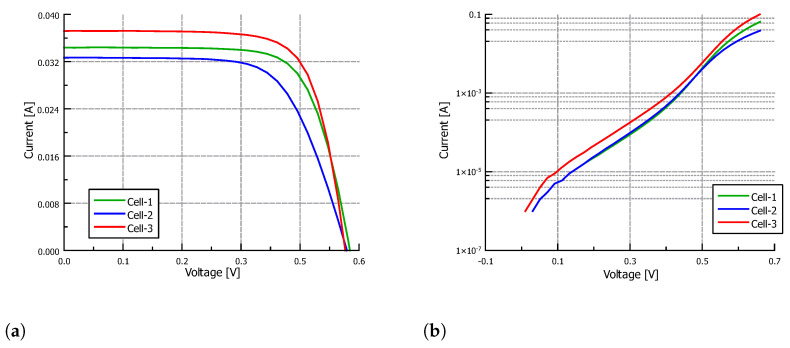
Experimental (**a**) illumination and (**b**) dark I-V characteristics of the fabricated solar cells.

The proposed algorithm is used in this section to extract the parameters of silicon solar cells fabricated at the National Institute for Astrophysics, Optics and Electronics. The cells are $1\;{\mathrm{cm}}^{2}$, and their structure is shown in Figure 6. They were fabricated using Czochralski Silicon P-type (100) wafers with 300μm thickness and typical resistivity of 5–15 Ω-cm. First, the wafers were cleaned and degreased with trichloroethylene and acetone, and the native oxide was removed in a hydrofluoric acid buffered solution BHF (7:1). Next, the standard cleaning processes RC1 and RC2 were carried out. After that, the wafers were immersed into an etchant solution (potassium hydroxide/isopropyl/isopropyl alcohol/deionized water) for 50 min in order to texturize them. The emitter (n+) was diffused, and the pre-deposition was carried out at 950 ${}^{\circ }$C for 10 min in a mixture of phosphine (PH3), nitrogen (N2) and oxygen (O2), while the re-diffusion was carried out for 10 min at 950 ${}^{\circ }$C using wet oxidation. Next, by photolithography, superficial windows were opened through superficial SiO2, and the aluminum (Al) contacts were formed by electron-beam evaporation and the lift-off technique. The only difference in the fabrication of the three solar cells considered in this section was in the final process steps, when the back oxide was removed and aluminum was used to create the Back Surface Field (BSF) and back contact. In the first case (Cell-1), an aluminum film was evaporated with a thickness of 5μm and, subsequently, Rapid Thermal Annealing (RTA) at 650 ${}^{\circ }$C for 180 s was carried out. In the second case (Cell-2), aluminum paste was used in order to obtain a film with a thickness of 20μm, after which an RTA was performed at 850 ${}^{\circ }$C for 180 s. Finally, in the third case (Cell-3), an RTA at 650 ${}^{\circ }$C for 120 s was performed after evaporation of a 5μm thick aluminum film [39,40]. The solar cells were characterized both at Standard Test Conditions (STC) and in the dark. Figure 7a,b show, respectively, their illumination I-V and dark I-V characteristics.

**Figure 8:**
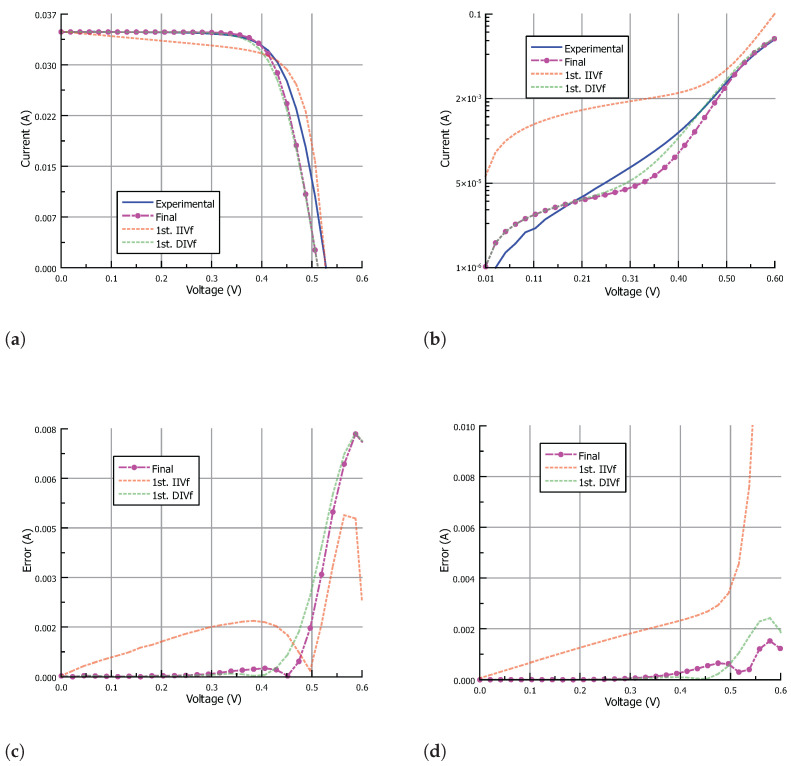
The I-V characteristic approximation of Cell-1 (**a**) under illumination and (**b**) in the dark; and corresponding absolute error (**c**) under illumination and (**d**) in the dark. Results are shown for parameters obtained after the first IIVf subroutine, after the first DIVf subroutine, and at the end of the parameter extraction process.

**Figure 9:**
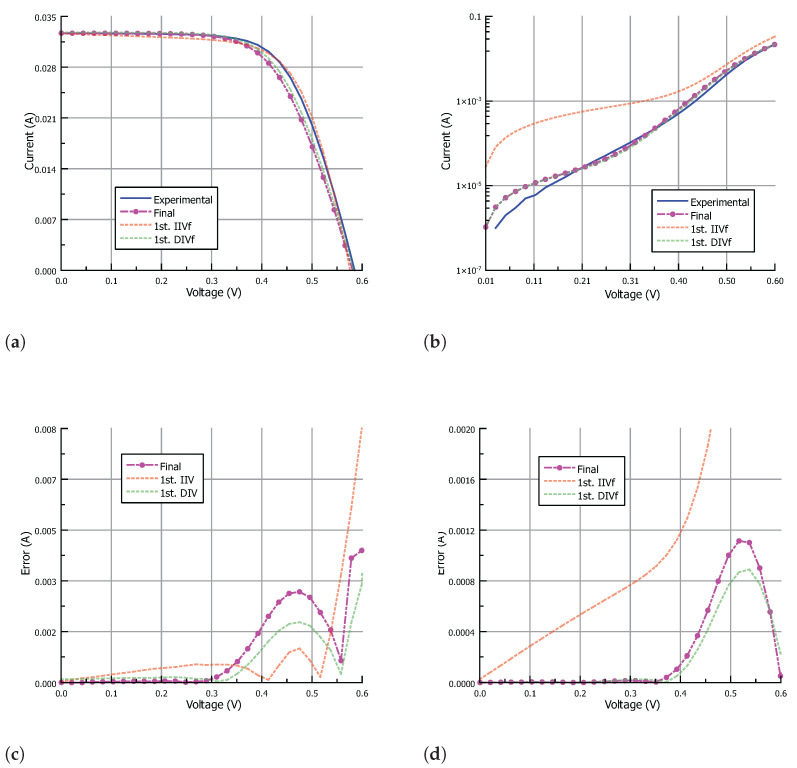
The I-V characteristic approximation of Cell-2 (**a**) under illumination and (**b**) in the dark; and corresponding absolute error (**c**) under illumination and (**d**) in the dark. Results are shown for parameters obtained after the first IIVf subroutine, after the first DIVf subroutine, and at the end of the parameter extraction process.

**Figure 10:**
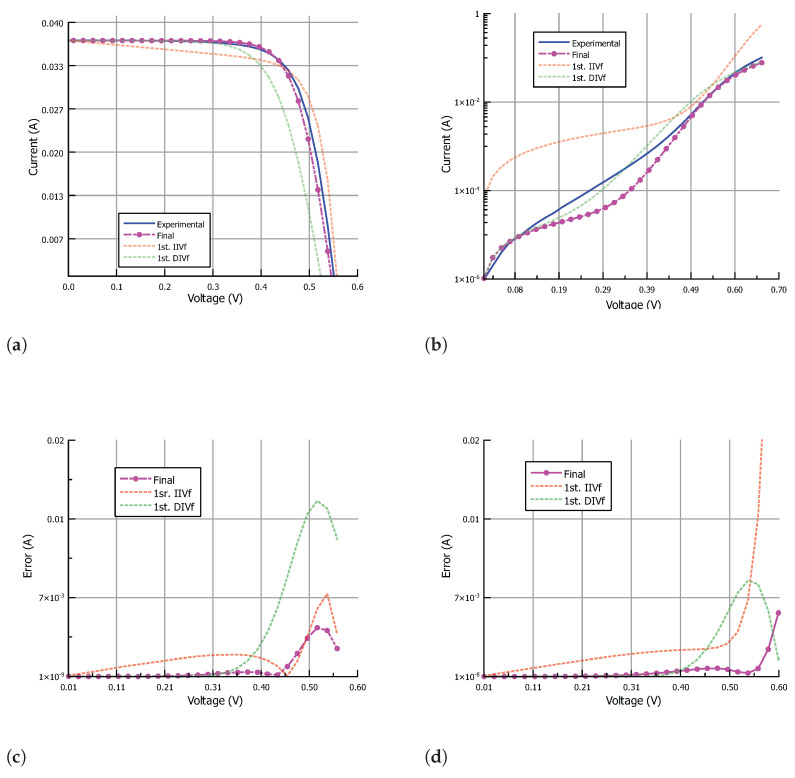
The I-V characteristic approximation of Cell-3 (**a**) under illumination and (**b**) in the dark; and corresponding absolute error (**c**) under illumination and (**d**) in the dark. Results are shown for parameters obtained after the first IIVf subroutine, after the first DIVf subroutine, and at the end of the parameter extraction process.

Table 2 shows the input data for the IIVf subroutine obtained from the IIV characteristic. The stop condition for the IIVf subroutine was set to a maximum error in $P_{max}$ of 1% while keeping the mean error in the output current lower than 0.1mA. The stop condition for the DIVf subroutine was set to a maximum mean error value in the output current of 0.1mA. Finally, the algorithm ends when the difference between the parameter values from subsequent IIVf and DIVf subroutines is minimum. In particular, the following stop conditions were set: the change in $R_{s}$ should be lower than 100mΩ, the change in $R_{sh}$ should be lower than 0.1kΩ, the change in $I_{si}$ (with i=1,2) should be lower than a factor of 2, and the change in $n_{i}$ (with i=1,2) should be lower than 0.05. Figure 8, Figure 9 and Figure 10 show the results of the fitting process under illumination and in the dark, and the corresponding absolute error, for the parameters obtained after the first IIVf subroutine, after the first DIVf subroutine, and at the end of the parameter extraction process. After the first IIVf subroutine, the maximum error in $P_{max}$ is lower than 1% in all cases, as established by the stop condition. However, using the obtained parameters to extract the dark I-V characteristic results in poor accuracy, and the need for further parameter adjustment is clear. As an example of how the parameter values evolve in the fitting process, Table 3 shows the extracted parameters of Cell-3 in the first and last iteration of the process, as well as the RMSE values in order to evaluate each fitting curve. In the last iteration, the difference between the parameter values meets the aforementioned stop conditions, and the algorithm ends. From the two sets of parameters obtained in the last iteration, the final set is chosen to be the one which results in the lowest mean value of RMSE. In this example case, the final parameter set stems from the last IIVf subroutine.

Table 4 shows the extracted parameter values for each solar cell. Good fitting is achieved in all cases, with values of RMSE lower than 0.006A and a maximum error in $P_{max}$ lower than 0.7%. Finally, it is worth mentioning that the extraction of parameters of the DDM taking into account the physics of the semiconductor devices allows process engineers to identify the bottlenecks associated with their solar cell manufacturing process, so they can improve the operation and performance of the PV cells by modifying and changing the physical and opto-electronic properties of materials (thickness, refractive index, doping, etc.), as well as the steps of the process fabrication itself. The results in Table 4, for example, show that the parameters of the cells vary with the RTA process that forms the BSF and back contact. In particular, lower Rs, $n_{1}$ and $n_{2}$ are obtained for Cell-1 and Cell-3, for which the RTA was performed at lower temperature (650 ${}^{\circ }$C vs. 850 ${}^{\circ }$C). From these devices, Cell-3, for which the RTA was performed for less time (120 s vs. 180 s), shows the best performance, with the lowest $R_{s}=1.184\;\Omega$, $n_{1}=1$ and $n_{2}=1.3$. However, in order to obtain the highest efficiency, the series resistance in monocrystalline cells should be kept lower than $0.5\;\Omega {\mathrm{cm}}^{2}$[41], i.e., lower than 500mΩ for $1\;{\mathrm{cm}}^{2}$ PV devices. The obtained parameters show that $R_{s}$ does not meet this condition in the fabricated solar cells, so future work should be focused on further reducing the ohmic resistance of contacts. In contrast, the condition that the shunt resistance should be greater than $1000\;\Omega {\mathrm{cm}}^{2}$ [41] is met in all three cases.

Table 5 shows a comparison with other parameter extraction methods. They rely either only on the illumination I-V characteristic or only on the dark I-V characteristic. The models are compared in terms of the accuracy in the prediction of the maximum power in the case of the light I-V curve adjustment, which was the parameter established as the stop condition for the IIVf subroutine, and in terms of $R^{2}$ and the $RMSE_{log_{10}I}$ in the case of the dark I-V curve, which are the figures of merit provided by the authors. Note that even though curve fitting is better in other cases, they lack the main contribution of the proposed method: providing good accuracy in the prediction of the maximum power point while ensuring that the resulting parameters keep their physical meaning by simultaneously fitting the dark I-V characteristics. To the best of our knowledge, no other parameter extraction algorithm considers both I-V curves.

## 4. Conclusions

A parameter extraction method for solar cells using the double-diode model is proposed in this paper. It fits both the illumination and the dark I-V characteristics, thus resulting in meaningful parameter values which can not only predict the MPP but also provide information about the physical performance of the PV devices. The algorithm consists of two subroutines which are alternatively run to adjust all the parameters of the cell in an iterative process. The illumination I-V fitting subroutine starts with an initialization step based on simplified equations to estimate the values of the parameters from three experimental points: the open-circuit voltage, the short-circuit current and the maximum power point. Then, the iteration process is carried out to adjust all the parameters so as to reduce the fitting error in the MPP. The resulting values are fed into the dark I-V fitting subroutine, which divides the I-V curve into four voltage ranges where different parameters are dominant. Again, their values are adjusted in an iterative way, and the results are fed back into the first subroutine. The process is repeated until the difference between the parameters obtained from subsequent subroutines is minimum, and the valid parameter set is the one which better fits both characteristics according to the RMSE value. The proposed method can be used to extract the parameters of any photovoltaic device, either commercial or fabricated in research laboratories, as long as their operation relies on the p–n junction. In this paper, the algorithm is validated by extracting the parameters of three in-house monocrystalline silicon solar cells with the same structure but subject to different temperature and time conditions during the Rapid Thermal Annealing process to form the back surface field and the back contact. The parameter set found for each cell results in good fitting of both illumination and dark I-V characteristics, with RMSE lower than 0.006 A in all cases and a maximum error in the maximum power of 0.69%. Furthermore, the resulting parameter values provide information about where to focus future efforts in order to improve the performance of the fabricated solar cells.

## Figures and Tables

**Table 1:** Summary of the behavior of I-V curve.

Parameter Variation	$P_{\max}$ Variation
$R_{s}$↑ (↓)	↓ (↑)
$R_{sh}$↑ (↓)	↑ (↓)
$n_{1}$ and $n_{2}$ ↑ (↓)	↑ (↓)
$I_{s1}$ and $I_{s2}$ ↑ (↓)	↓ (↑)

**Table 2:** Experimental values from IIV of solar cells.

Parameter	Cell-1	Cell-2	Cell-3
$I_{sc}$ (mA)	34.39	32.67	37.19
$V_{oc}$ (mV)	558	558	558
$I_{mpp}$ (mA)	31.69	28.70	34.23
$V_{mpp}$ (mV)	436	416	436

**Table 3:** Evolution of the extracted parameters for Cell-3.

Parameter	First Iteration		Final Iteration	
	IIVf	DIVf	IIVf	DIVf
$R_{s}$ (Ω)	0.039	1.336	1.184	1.214
$R_{sh}$ (kΩ)	0.1	10.1	10.0	10.0
$I_{s1}$ (pA)	50.3	12.9	4.02	5.85
$I_{s2}$ (nA)	7.12	64.4	2.01	2.92
$n_{1}$	1.1	1.2	1.0	1.0
$n_{2}$	1.5	1.6	1.3	1.3
IIV RMSE (A)	0.0915	0.00736	0.001353	0.00662
DIV RMSE (A)	0.0963	0.00509	0.005224	0.00522
$P_{max}$ error (%)	0.53	10.9	0.07	3.8

**Table 4:** Model parameter values.

Parameter	Cell-1	Cell-2	Cell-3
$I_{pv}$ (mA)	34.39	32.67	37.19
$R_{s}$ (Ω)	1.707	2.854	1.184
$R_{sh}$ (kΩ)	9.9	10.0	10.0
$I_{s1}$ (pA)	40.8	274	4.02
$I_{s2}$ (nA)	5.23	40.8	2.01
$n_{1}$	1.1	1.3	1.0
$n_{2}$	1.8	1.6	1.3
IIV RMSE (A)	0.002660	0.001289	0.001353
DIV RMSE (A)	0.001349	0.000624	0.005224
$P_{max}$ error (%)	0.68	0.53	0.07
Iterations	6	3	7

**Table 5:** Comparison with other parameter extraction methods.

	Solar Cell	Light I-V	Dark I-V	
		$P_{\max}$ Error (%)	${\mathrm{RMSE}}_{\log_{10}I}$	$R^{2}$
Orioli [35]	Poly & mono crystalline	<0.27	-	-
Tifidat [32]	Poly & mono crystalline	<0.002	-	-
Hallam [13]	Poly & mono crystalline	-	<0.14	-
Macabebe [11]	monocrystalline	-	-	>0.991
Proposed	monocrystalline	<0.68	<3.37	>0.995
